# *Klotho* null mutation leads to retinal degeneration characterized by functional impairment, gliosis, and deposition of amyloid-beta and hyperphosphorylated tau proteins

**DOI:** 10.1371/journal.pone.0323633

**Published:** 2025-05-15

**Authors:** Zhi-Jia Chen, Chun-Yen Wu, Fang-Yan Hsiao, Jing-Chun Ma, Gabriel Lai, Han-Hsin Chang, David Pei-Cheng Lin

**Affiliations:** 1 Department of Medical Laboratory and Biotechnology, Chung Shan Medical University, Taichung, TaiwanRepublic of China; 2 Department of Nutrition, Chung Shan Medical University, Taichung, TaiwanRepublic of China; 3 Department of Ophthalmology, Chung Shan Medical University Hospital, Taichung, TaiwanRepublic of China.; Georgetown University Medical Centre, UNITED STATES OF AMERICA

## Abstract

**Background:**

*Klotho* mutation has been known to accelerate aging and degenerative pathogenesis, notably in the kidney and the brain. Nevertheless, the aftermath of *Klotho* function deprivation in the retina has not been detailed. This study aimed to provide an in-depth analysis of retinopathy caused by *Klotho* mutation.

**Results:**

The homozygous *Klotho* mutant mice retinas were analyzed at the 6^th^, 8^th^ and 10^th^ weeks of age, along with their heterozygous, and wild-type littermates between both genders. The electroretinogram results showed retinal function impairment with *Klotho* null mutation, as compared with their littermates. Nevertheless, there was no difference in the retinal layer thickness, morphology, and cell death up to 10 weeks of age among the three genotypes. No evident damage was detected in the photoreceptors, interneurons, and retinal ganglion cells with *Klotho* mutation up to 10 weeks of age. Amyloid-beta and hyperphosphorylated tau protein deposits were detected in the *Klotho* mutant retina, along with glial cell activation at 10 weeks of age.

**Conclusions:**

The results revealed that *Klotho* null mutation leads to retinal degeneration characterized by functional impairments, gliosis, and amyloid-beta and hyperphosphorylated tau protein deposition in the retina. Klotho protein function is, therefore, mandatory for the maintenance of a healthy retina. The mouse *Klotho* null mutation may be used as a study model for age-related retinal degeneration and Alzheimer’s disease.

## Introduction

*Klotho* is identified as an anti-aging gene that can quench several aging phenotypes, including shortened lifespan, ectopic calcification, osteoporosis, arteriosclerosis, and skin atrophy, among others [1]. The average lifespan of *Klotho* mutant mice is about 8 weeks, with a maximum of less than 14.2 weeks [1]. The mice with *Klotho* overexpression can extend their lifespan by 20–30% longer than the wild-types [2]. As Klotho protein can be detected in the retina, it is also believed to contribute to several beneficial effects including the promotion of phagocytosis, melanogenesis, inhibiting VEGF secretion, maintaining cellular organization and homeostasis of the retina, and preserving retinal function or resistance to oxidative stress via various signaling cascades [3,4]. Furthermore, *Klotho* could impede subretinal fibrosis and choroidal neovascularization (CNV) and reverse these pathologies when overexpressed [5]. Several studies demonstrated the protective effects of *Klotho* in Alzheimer’s disease (AD) patients. AD, prevalently diagnosed in the elderly as a form of mental failure [6–8], is characterized by deposits of amyloid-beta (Aβ) plaques and formation of neurofibrillary tangles (NFTs) which are primarily composed of hyperphosphorylated tau (pTau) in the brain. *Klotho* overexpression can attenuate AD-like cognitive impairments by eliminating Aβ via autophagy, inhibiting pTau accumulation, and reducing neuronal and synaptic loss [9,10]. Also, *Klotho* is reported to ameliorate the release of inflammatory cytokines in the AD patients [11–13].

Aside from cognitive abnormalities, AD patients also suffer from ophthalmologic disorders, including impaired functions in visual acuity, color vision, and contrast sensitivity; and all these have been reported in the early stage of AD patients [14]. These ophthalmologic dysfunctions are also associated with the accumulation of Aβ and pTau in the retina of AD patients [14–17]. On the part of Aβ, *in vitro* cell line studies have reported that retinal ganglion cell (RGC) death and retinal pigmented epithelium (RPE) senescence were induced by elevated levels of Aβ [18,19]. Moreover, an animal model of AD (bitransgenic APP/PS1 mouse) was characterized by Aβ accumulation not only in the brain, but also in the inner plexiform layers (INL), nerve fiber layer (NFL), and ganglion cell layer (GCL) retinal layers where apoptosis was colocalized [20].

As for pTau, growing evidences have indicated its accumulation that lead to retinal degeneration [21], as examplified by a clinical study showing pTau deposits in the outer plexiform layers (OPL) and inner plexiform layers (IPL) in the retina of AD patients [22]. In the mice with transgenic human P301S tau, pTau was accumulated in the NFL and RGC [23]. Conversely, treatment with N-terminal tau 12A12 monoclonal antibody in the AD-like animal model (Heterozygous female Tg2576 mice) led to reduced pTau quantity in the retina, concomitant with mitigation of inflammation, abnormal neurochemical and neuronal variations, and curbing of apoptosis [24]. These data suggest Aβ and pTau are not only the hallmarks in the AD brain but also play a crucial role during the neurodegenerative process in the retina of AD patients.

Glia cells provide various functions, e.g., removing metabolic waste products and microbes, providing nutrients, and regulating immune response, to maintain the retina health [25]. Glia cells consist of Müller, astrocytes, and microglial cells [26]. The somata of Müller cells are located in the INL, and Müller cells span radially from the inner limiting membrane (ILM) to the outer limiting membrane (OLM) [27]. Astrocytes reside in the GCL and NFL. Microglia cells are distributed in the IPL and OPL of the retina [28]. Upon retinal damage, Müller cells and astrocytes become activated and express higher levels of glial fibrillary acidic protein (GFAP) [29]. The microglia cells are activated to proliferate and transform into an ameboid morphology to enable migrating to the damaged site [30,31]. These microglial behaviors can be visualized and traced using the microglia cell marker Iba-1 [25,28,30]. Nevertheless, excessively activated glial cells may be out of control, resulting in negative effects and even deteriorating the damage to the retina [26,30], as suggested by the presence of activated glial cells during the pathogenesis of AD [32–34].

Based on the afore-mentioned information, the AD patients and AD animal models exhibit Aβ and pTau deposition as well as gliosis, and a connection between *Klotho* and AD has been established. However, there has been limited research on the effect of *Klotho* mutation related to AD pathology in the retina. This study aimed to investigate the abnormalities in the *Klotho* mutant retina and to elucidate whether the *Klotho* mutation may exhibit AD-like pathology in the retina.

## Materials and methods

### *Klotho* mutant mice breeding and genotyping

The *Klotho* mutant mice with genetic background on C57BL/6J were obtained from Mutant Mouse Resource & Research Center (MRRC), UC Davis, USA. The strain was maintained in the National Laboratory Animal Center (NLAC), Taiwan, and transferred to Chung Shan Medical University, Taichung City, Taiwan. The mice were bred in individually ventilated cages and maintained at 20°C to 24°C with 50% to 55% humidity under a 12-hour light/12-hour dark cycle. All commercial diets and water were sterilized by an autoclave and given to the mice *ad libitum*.

All animal care and experimental procedures were performed according to the standard laboratory animal protocols that had been approved and licensed by the Institutional Animal Care and Use Committee of Chung Shan Medical University (IACUC Approval No:2472) in accordance with the ARVO Statement for the Use of Animals in Ophthalmic and Vision Research.

The mouse genotypes were confirmed by PCR amplification based on a protocol developed by the Mutant Mouse Resource & Research Center (MMRRC) at the University of California, Davis. Two sets of primers as following were used: 5’- GATGGGGTCGACGTCA-3’ (forward for *Klotho*) and 5’- TAAAGGAGGAAAGCCATTGTC-3’ (reverse for *Klotho*) for 186 bp amplicon to indicate wild-type; 5’-GCAGCGCATCGCCTTCTATC-3’ (forward for *Klotho* mutant) and 5’-ATGCTCCAGACATTCTCAGC-3’ (reverse for *Klotho* mutant) for 455 bp amplicon to indicate mutant genotype. The PCR parameters were set as: 5 minutes for initiation at 94 ^o^C. Then 35 cycles of 15 seconds for denaturation at 94 ^o^C, 15 seconds for annealing at 65 ^o^C (for *Klotho* mutant) and 55 ^o^C (for wild-type), and 30 seconds for elongation at 72 ^o^C followed by a final amplification at 72 ^o^C for 5 minutes. The PCR products were preserved at 10 ^o^C in the end. Obtainment of both 186 bp and 455 bp amplicon was regarded as heterozygotes, only 186 bp amplicon as wild-types, and only 455 bp amplicon as homozygotes.

### Electroretinogram (ERG)

Experimental mice were divided in to different groups in accordance to 3 genotypes (wild-type, heterozygote, and mutation) and 3 ages (6 weeks, 8 weeks, and 10 weeks of age), and then were dark-adapted for 12 hours prior to the experiment. Mice were anesthetized with an intraperitoneal injection of Zoletil50® (50 mg/ml; Suffolk, UK) and Rompun® (23.32 mg/ml; Leverkusen, Germany). After anesthetization, mice were placed in the dry bath incubator at 32°C. The cornea was applied with MYDRIACYL® (Alcon, Fort Worth, TX, USA) and another eye was applied with Tears Naturale Artificial Tears (Alcon, Fort Worth, TX, USA). Next, the cornea used for the ERG test was anesthetized with ALCAINE 0.5% (Alcon, Fort Worth, TX, USA). The red, white, and black wire electrode were carefully placed on the cornea, eyelid, and tail, respectively. ERG was recorded at the beginning of non-light stimulus for 60 seconds, followed by light stimulation with 120 times/min frequency and 5 cd*s/m^2^ intensity for 60 seconds, and keep this order in a similar fashion for 3 times. ERG was recorded by BIOPAC MP36 hardware (Goleta, CA, USA) and the graph of ERG recording was analyzed by Biopac Student Lab 4.1 software (Goleta, CA, USA).

### Tissue processing

The samples from the study groups were collected, according to 3 genotypes (wild-type, heterozygote, and mutation) and 2 ages (8 weeks, 10 weeks of age).

For euthanasia, each mouse was placed into a transparent chamber infused with 100% CO2 at a rate of 20% displacement of the chamber volume per minute. The entire procedure was completed in 5 minutes. Once the mouse appeared unconscious with no sign of cardiac beat and respiration and showed dilated pupils, the euthanasia was considered completed. At the 8, and 10 weeks of age, the sample eyes were enucleated carefully from the euthanized mice, and then immersed in Davidson’s solution for at least 24 hours for fixation. Afterwards, the eyes were dehydrated and processed to imbed in the paraffin. 5 µm serial sections were cut using a Semi-Automatic Rotary microtome (RWD life science; Minux® S700) and allowed to dry at 60°C.

### Histological detection

Hematoxylin and eosin staining of retinal sections was conducted via deparaffinization, rehydration, and staining in Mayer’s Hematoxylin (Dako, cat. no. S3309) for 100 seconds. Next, the slides were washed 3 times and de-stained with acidic alcohol for 10 seconds, followed by staining with 3% Eosin Y (Sigma cat. no. E6003). After being completely air-dried, the slides were mounted by coverslips with Micromount (Leica, cat. no. 3801731) and photographed at 100x for quantification of each layer in the retina and at 400x for representative images under a light microscope (Leica M500-N).

### TUNEL (Terminal deoxynucleotidyl transferase dUTP nick end labeling) assay

For detection of apoptotic cells in the retina, deparaffined and rehydrated retinal sections were stained with by One-step TUNEL In Situ Apoptosis Kit (Red, Elab Fluor® 555) (Elabscience, E-CK-A325, Houston, Texas, USA) in accordance to the manufacturer’s protocol.

All images were taken at the identical exposure time for every group. Only the cells in the retina with double staining of TUNEL and DAPI were considered as TUNEL-positive cells. TUNEL-positive cells counting was manually performed from the optic nerve head (ONH) to the retinal margin along the superior and inferior halves. During the counting the TUNEL-positive cells, background noise and autofluorescence were excluded by referencing the negative control to ensure the accuracy and the specificity (S1 Fig).

### Immunohistochemistry (IHC)

For IHC on retinal paraffin sections, the sections were deparaffinized and rehydrated (10 minutes for 3 times in xylene, 10 minutes for 3 times in 100% ethanol, 1 minute for 1 time in 95% ethanol, 1 minute for 1 time in 75% ethanol, 1 minute for 1 time in 50% ethanol, 1 minute for 1 time in 25% ethanol, and 1 minute for 2 times in double distilled water). After that, antigen retrieval was performed through microwave heating for 25 mins within citrate buffer (pH = 6.0). The slides were then allowed to slowly cool to room temperature, washed in running tap water, and endogenous peroxidase blockage was performed in a 5% hydrogen peroxide solution, followed by washing in the tris-buffer saline (TBS) and immersion in a 1% bovine serum albumin (BSA) solution for 10 minutes to prevent nonspecific interactions. Retinal sections were incubated in the following primary antibodies diluted in the 1% BSA solution: mouse anti-rhodopsin (1:400; Abcam ab81702), rabbit anti-opsin (1:300; Novus Biologicals NB110–74730), and rabbit anti-calbindin (1:500; Abcam ab108404), rabbit anti-Tuj1 (1:400; Novus Biologicals NB110–57611). The retinal sections were incubated with primary antibodies in a humid box at 4°C overnight. The retinal sections were then rinsed with TBS three times for 5 mins, and then incubated with the rabbit secondary antibody (cat. No. 305035003; Jackson ImmunoResearch Laboratories) diluted to 1:200 with a 1% solution of BSA, and the slides were kept in a humid box at 4°C overnight. The preparations were then washed by TBS at room temperature (RT). For color detection, 3,3′- diaminobenzidine (DAB, 40 mg/dL; DAKO, USA) was added to the retinal sections for 5 mins. Afterwards, the retinal sections were counterstained with hematoxylin, air-dried, and mounted by coverslips with Micromount (Leica, cat. no. 3801731). The images were captured at 100x for quantification of targeted signals and 400x for representative images under a light microscope (Leica M500-N).

Fluorescence detection was also performed. To detect the fluorescent signals, the primary antibodies, including, mouse anti-amyloid beta (1:200; Novus Biologicals NBP2-13075), rabbit anti-pTau (1:200; Novus Biologicals NBP2-67702), rabbit anti-GFAP (1:300; Sigma-Aldrich SAB2107063), and rabbit anti-Iba-1 (1:400; Abcam AB178846) were diluted with 1% solution of BSA. The incubations were kept in a humid box at 4°C overnight. The sections were then rinsed with TBS three time for 5 mins and the primary antibodies was detected with relevant secondary antibodies labeled with donkey anti-Alexa Fluor® 594 (1:400; Abcam ab150108) or goat anti-Alex Fluor® 488 (1:400; Abcam ab150077). The cell nuclei staining was performed by incubation at RT for 5 min by DAPI (product No. D9542; Sigma-Aldrich). The sections were mounted in Micromount (Leica, cat. no. 3801731) after a complete airdry. The images were photographed at 200x for representative images and quantification of targeted signals in the retina by a fluorescent microscope (Axio Imager A2; ZEISS).

### Quantification of retinal thickness and immunohistochemical intensity measurement

Retinal thickness measurement was manually performed at 400 μm, 800 μm, 1200 μm, and 1600 μm intervals to the ONH along the superior and inferior halves. The thickness of retinal pigmented epithelium (RPE), outer segment (OS), inner segment (IS), outer nuclear layer (ONL), outer plexiform layer (OPL), inner nuclear layer (INL), inner plexiform layer (IPL), ganglion cell layer (GCL), nerve fiber layer (NFL), OS to GCL, RPE to GCL, and RPE to NFL layers was measured at each defined point for every group by Image J (National Institutes of Health, an open-source image processing software) [35].

For intensity measurements of opsin in OS/IS and rhodopsin in OS in DAB detection, Image J was applied as conducted in a previous protocol [36]. Opsin and rhodopsin intensity measurement was manually performed from the ONH to the retinal margin along the superior and inferior halves. Calbindin-positive cells in INL and Tuj1-positive cells in GCL were counted within 400 μm intervals to ONH along the superior and inferior halves by Image J. All DAB staining immunohistochemistry images were taken at the identical exposure time and other parameters for every group.

For fluorescent intensity measurements, Image J was applied as conducted according to a previous protocol [37]. All images were taken at the identical exposure time for every group. Images were split into RGB channels. The intensity of region of interest (ROI) of single channel that corresponded to the secondary antibody fluorochrome was converted to the integrated density of pixel value falling range of threshold value based on the nature of the image and fluorescence intensity. Fluorescent signal measurement was manually performed from the ONH to the retinal margin along the superior and inferior halves. During the measurement of the target fluorescent signals, background noise and autofluorescence were excluded by referencing the negative control to ensure the accuracy and the specificity (S1 Fig).

### Statistical analysis

The data of all experimental groups were analyzed using the GraphPad Prism v9. (GraphPad Prism version 10.0.0 for Windows, GraphPad Software, Boston, Massachusetts USA) The experimental values among of wild-type, *Klotho* heterozygote, and *Klotho* mutation at 6^th^, 8^th^, and 10^th^ weeks of age were analyzed by the one or two-way-ANOVA (or mixed model) with Bonferroni multiple comparisons test. A significant difference was set when the *p-value* was less than 0.05. p**, p***, and p**** were used to represent less than 0.01, 0.001, and 0.0001, respectively.

## Results

### *Klotho* mutation lead to impaired ERG display

In this study, we utilized the ERG technique to record the retina reaction in response to light stimulation [38]. The scotopic (rod pathway) ERG response among the wild-type, *Klotho* heterozygous, and *Klotho* mutant mice was recorded at 6, 8, and 10 weeks of age after being dark-adapted.

**Fig 1:**
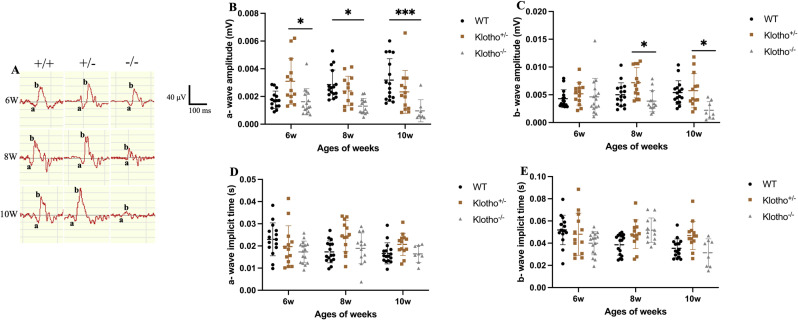
Representative scotoptic ERG results and comparison among the three genotypes at 6, 8 and 10 weeks of age. (A) Representative scotoptic ERG with annotation of a- and b-wave at 120 times/min frequency and 5 cd*s/m^2^ intensity from dark-adapted mice among the three genotypes at 6, 8 and 10 weeks of age. (B–E) Comparison of a- and b-wave amplitude (mV) and implicit time (s) among the three genotypes at 6, 8, and 10 weeks of age. A- and b-wave amplitude were measured from baseline to trough and from baseline to peak, respectively. Implicit time were presented as time to peak/trough. Scatter plots show the means and standard deviations. Significant difference was determined by two-way ANOVA with Bonferroni multiple comparisons test, and presented as *p < 0.05, **p <0.01, ***p <0.001, respectively.

We observed a significantly reduced average a-wave amplitude in *Klotho* mutant mice compared to *Klotho* heterozygous mice at 6 weeks of age (Fig 1A and B, Table 1). Additionally, average a-wave amplitude in *Klotho* mutant mice decreased significantly compared to wild-type mice at 8 and 10 weeks of age (Fig 1A and B, Table 1). *Klotho* mutant mice also exhibited significantly lower b-wave amplitude than *Klotho* heterozygous mice at 8 and 10 weeks of age (Fig 1A, C, Table 1). There were no significant differences in a and b- waves implicit time among the wild- type, *Klotho* heterozygous, and *Klotho* mutant mice at 6, 8, and 10 weeks of age (Fig 1A, D, E, Table 1).

**Table 1:** Summary of ERG results among the three genotypes at 6, 8, and 10 weeks of age.

Genotype	WT	Klotho^ + /-^	Klotho^-/-^	WT	Klotho^ + /-^	Klotho^-/-^	WT	Klotho^ + /-^	Klotho^-/-^
Sample sizes	15	13	16	16	13	13	16	13	8
Weeks of age	6	6	6	8	8	8	10	10	10
a-wave amplitude (mV)	0.00176	0.00309^b*^	0.00162^b*^	0.00285^c*^	0.00239	0.00130^c*^	0.00318^c**^	0.00233	0.00096^c**^
a-wave implicit time (s)	0.02296	0.01968	0.01731	0.01731	0.02439	0.01890	0.01665	0.02070	0.01642
b-wave amplitude (mV)	0.00429	0.00516	0.00463	0.00500	0.00717^b*^	0.00385^b*^	0.00539	0.00573^b*^	0.00220^b*^
b-wave implicit time (s)	0.05188	0.04801	0.03999	0.03857	0.04804	0.05171	0.03537	0.04690	0.03135

Significant difference was determined by two-way ANOVA with Bonferroni multiple comparisons test, and presented as *p < 0.05, **p < 0.01, ***p < 0.001. a indicated the comparison between WT and *Klotho*^+/-^, b indicated the comparison between *Klotho*^+/-^ and *Klotho*^-/-^, and c indicated the comparison between WT and *Klotho*^-/-^.

#### No difference in the retinal layer thickness and morphology among *Klotho* genotypes at 8 and 10 weeks of age.

**Fig 2:**
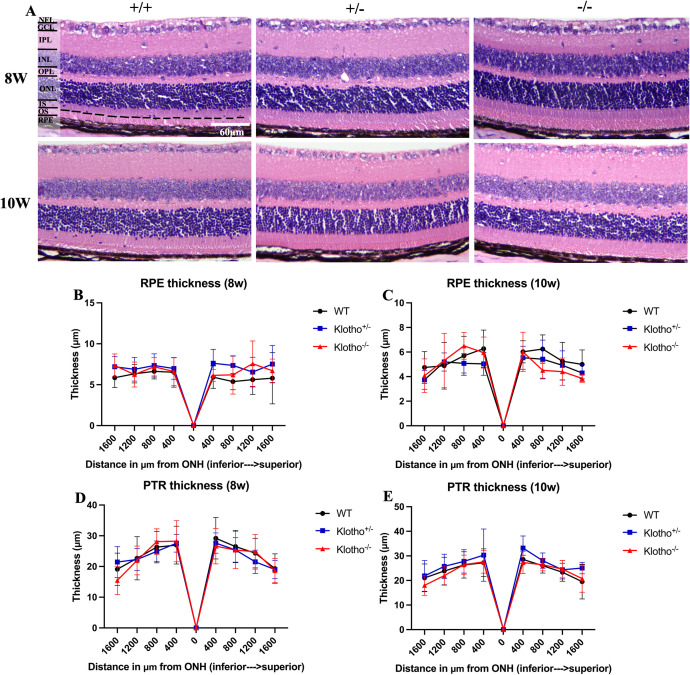
Representative images of Hematoxylin-eosin (H&E) staining and retina thickness analysis among the three genotypes at 8 and 10 weeks of age. (A) H&E staining images of the retina. The dash lines indicate the boundary between IS (inner segment) and OS (outer segment). (B–E) Comparison of the RPE (retinal pigmented epithelium) and PTR (photoreceptor) thickness among the three genotypes at 8 and 10 weeks of age. The thickness of RPE and PTR were measured at 400 μm, 800 μm, 1200 μm, and 1600 μm intervals away from the ONH (optic nerve head) and along the superior and inferior halves. n=6 per group presented as mean± SD. Significant difference was determined by two-way ANOVA with Bonferroni multiple comparisons test, and presented as *p < 0.05, **p < 0.01, ***p < 0.001.

We used H&E stain to examine the morphology and measure the retina thickness of the wild-type, *Klotho* heterozygous, and *Klotho* mutant mice at 8 and 10 weeks of age. There was no degeneration or disorganization in the retina of *Klotho* mutant mice at 8 and 10 weeks of age (Fig 2A).

Since the Klotho protein had been suggested to regulate the retinal pigmented epithelium function for the maintenance of the photoreceptors [3], we analyzed the thickness of RPE layer and photoreceptor layer. The results showed no significant difference in thickness of all measurements among the three genotypes at 8 and 10 weeks of age (Fig 2B–E). Besides, we also measured the thickness of ONL, OPL, INL, IPL, GCL, NFL, OS to GCL, RPE to GCL, and RPE to NFL among the three genotypes at the same age. No difference in these layers was found (S2 Fig). Moreover, no significant difference was observed in number of photoreceptor nuclei in the ONL and in cell death among the three genotypes at same age (S3 and S4 Figs).

#### No evident damage in the photoreceptors, interneurons and RGCs in *Klotho* mutant mice up to 10 weeks of age.

**Fig 3:**
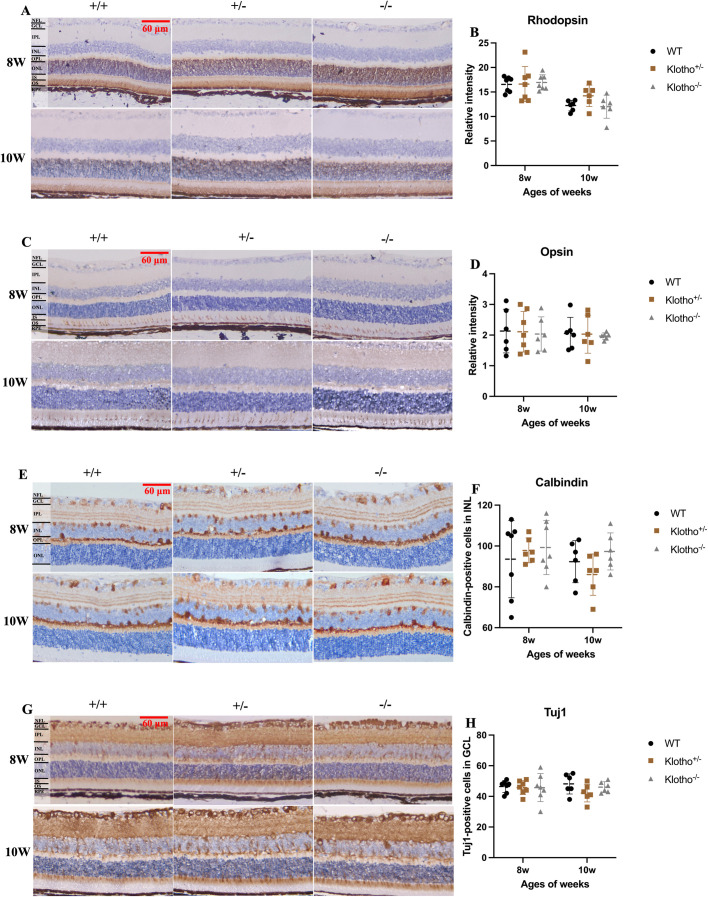
Representative images of immunohistochemical (IHC) staining and quantification of rhodopsin, opsin, Tuj1, and calbindin in the retina among the three genotypes at 8 and 10 weeks of age. (A) Rhodopsin IHC images. The brown color in the OS indicates the rhodopsin signals. (B) Quantification of rhodopsin in OS and comparison among the three genotypes at 8 and 10 weeks of age. (C) Opsin IHC images. The brown color in the OS/IS indicates the opsin signals. (D) Quantification of opsin in OS/IS and comparison among the three genotypes at 8 and 10 weeks of age. (E) Calbindin IHC images. The brown color of cells located in the INL indicates the calbindin-positive cells. (F) Quantification of calbindin-positive cells in INL and comparison among the three genotypes at 8 and 10 weeks of age. (G) Tuj1 IHC images. The brown color of cells located in the GCL indicates the Tuj1-positive cells. (H) Quantification of Tuj1-positive cells in GCL and comparison among the three genotypes at 8 and 10 weeks of age. Scatter plots show the means and standard deviations. Significant difference was determined by two-way ANOVA with Bonferroni multiple comparisons test, and presented as *p < 0.05, **p < 0.01, ***p < 0.001, respectively.

The rhodopsin and opsin are the main photoreactive proteins of the rod and cone photoreceptor cells, respectively [39,40]. Equivalent levels of rhodopsin and opsin expression of *Klotho* mutant mice was observed, as compared to those found in the wild-types and *Klotho* heterozygotes at the 8 and 10 weeks of age (Fig 3A–D). Tuj1 was used as a marker for RGC and calbindin was used as a marker for amacrine, horizontal, and bipolar cells located in the INL [4,41]. We found no difference in Tuj1 and calbindin expression levels among the three genotypes at the 8 and 10 weeks of age (Fig 3E–H).

#### Abnormal amyloid-beta (Aβ) and hyperphosphorylated tau (pTau) protein deposition in *Klotho* mutant retina.

**Fig 4:**
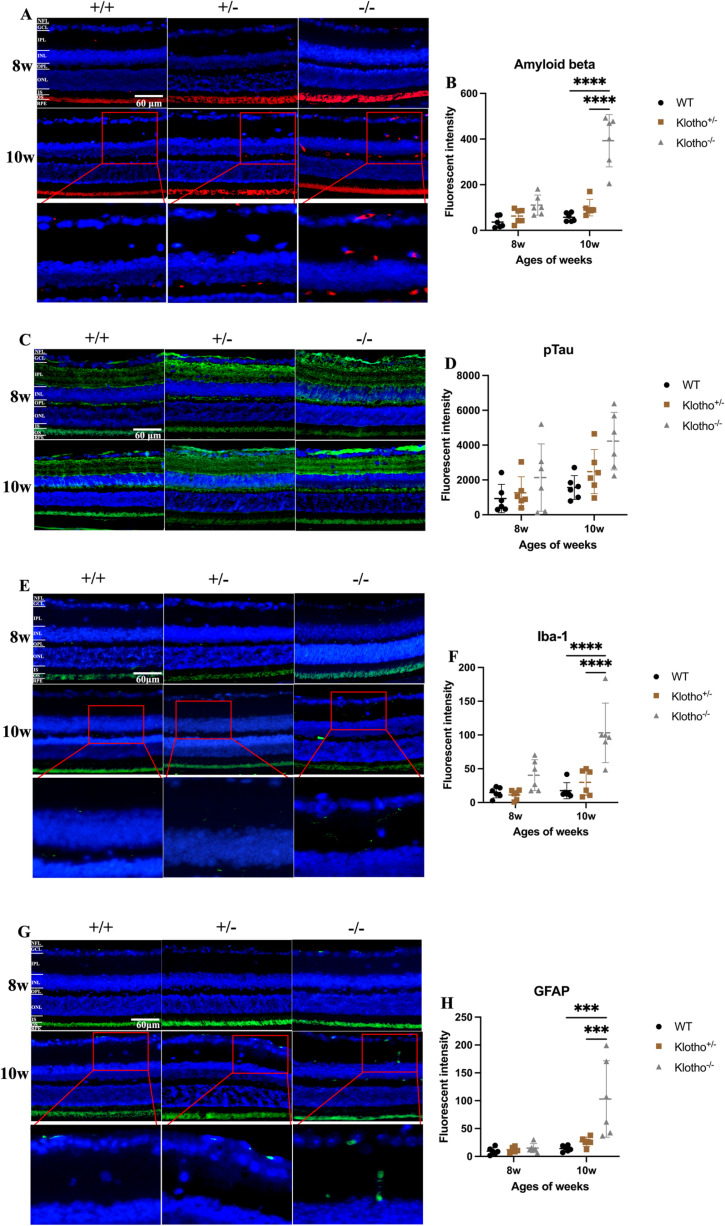
Representative images of immunofluorescent (IF) staining and quantification of A β, pTau, Iba-1, and GFAP in the retina among the three genotypes at 8 and 10 weeks of age. (A) IF images of Aβ (red signals) and magnification of Aβ signals. The blue dots are cell nuclei detected by DAPI. (B) Quantification of Aβ in the retina and comparison among the three genotypes at 8 and 10 weeks of age. (C) IF images of pTau (green signals). The blue dots are cell nuclei detected by DAPI. (D) Quantification of pTau in the retina and comparison among the three genotypes at 8 and 10 weeks of age. (E) IF images of Iba-1 (green signals) and magnification of Iba-1 signals. The blue dots are cell nuclei detected by DAPI. (F) Quantification of Iba-1 in the retina and comparison among the three genotypes at 8 and 10 weeks of age. (G) IF images of GFAP (green signals) and magnification of GFAP signals. The blue dots are cell nuclei detected by DAPI. (H) Quantification of GFAP in the retina and comparison among the three genotypes at 8 and 10 weeks of age. Scatter plots show the means and standard deviations. Significant difference was determined by two-way ANOVA with Bonferroni multiple comparisons test, and presented as *p < 0.05, **p < 0.01, ***p < 0.001, ****p < 0.0001 respectively.

To further elucidate the impaired ERG functions in the *Klotho* mutant mice, we performed immunostaining to examine whether Aβ and pTau were accumulated in the retina at 8 and 10 weeks of age. There was no significant difference in Aβ accumulation among the three genotypes at 8 weeks of age (Fig 4B). At 10 weeks, the *Klotho* mutant mice showed significantly more Aβ accumulation compared with the wild-type and *Klotho* heterozygotes (Fig 4B). Aβ accumulation was also located in the OPL, IPL, GCL, and NFL of the *Klotho* mutant mice at 10 weeks of age (Fig 4A). As for pTau deposition, the *Klotho* mutant mice did not show a significance increment at 8 and 10 weeks of age (Fig 4D). pTau was detected in IPL, GCL, and NFL of the *Klotho* mutant mice retina to an extent higher than that of the wild-type and *Klotho* heterozygotes at 8 and 10 weeks of age (Fig 4C).

#### Glia cell activation in the retina of *Klotho* mutant mice.

**Fig 5:**
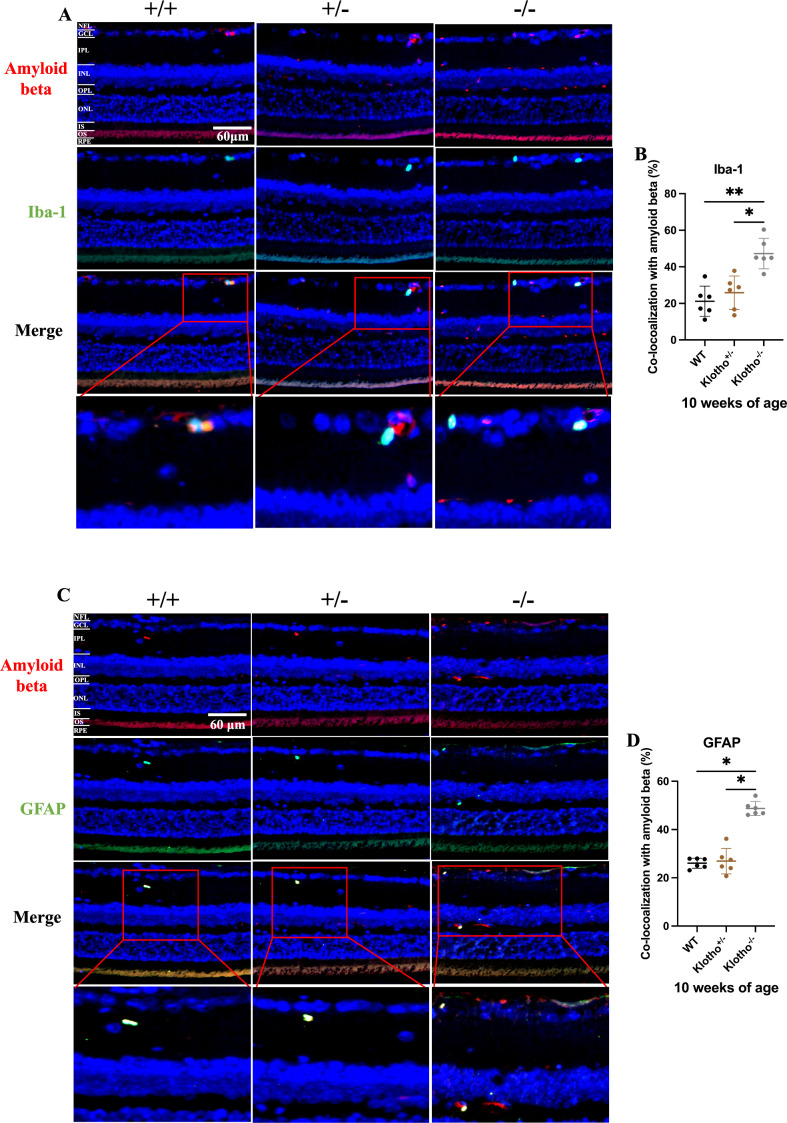
Representative images of immunofluorescent (IF) staining and quantification of co-localization analysis of A β with Iba-1 and GFAP in the retina among the three genotypes at 8 and 10 weeks of age. (A) IF images of Aβ (red signals), Iba-1 (green signals), Aβ and Iba-1 overlap, and magnification of Aβ and Iba-1 overlap. The blue dots are cell nuclei detected by DAPI. (B) Quantification of co-localization rate of Aβ and Iba-1 in the retina and comparison among the three genotypes at 8 and 10 weeks of age. (C) IF images of Aβ (red signals), GFAP (green signals), Aβ and GFAP overlap, and magnification of Aβ and GFAP overlap. The blue dots are cell nuclei detected by DAPI. (D) Quantification of co-localization rate of Aβ and GFAP in the retina and comparison among the three genotypes at 8 and 10 weeks of age. Scatter plots show the means and standard deviations. Significant difference was determined by one-way ANOVA with Bonferroni multiple comparisons test, and presented as *p < 0.05, **p < 0.01, ***p < 0.001, respectively.

To understand the aftermath of Aβ and pTau accumulation, we investigated the status of glial cell activation via immunodetection of GFAP and Iba-1. GFAP is a marker for astrocytes and Müller cells and is known to increase upon activation of both cells [42]. Iba-1 can be used to detect activation and visualize migration of microglia cells [25,30]. No significant difference was detected in GFAP and Iba-1 among the three genotypes at 8 weeks of age (Fig 4F, H). At 10 weeks of age, the *Klotho* mutant mice showed a significant increment of GFAP and Iba-1 expression compared with the wild-type and *Klotho* heterozygotes (Fig 4F, H). The activated microglia cells were found in the OPL, IPL, GCL, and NFL of the *Klotho* mutant mice at 10 weeks of age (Fig 4E). Besides, astrocyte activation was observed in OPL, IPL, GCL and NFL of the *Klotho* mutant mice at 10 weeks of age (Fig 4G). We also performed co-localization analysis of Aβ with Iba-1 and GFAP and found that the co-localization rates were significantly higher in *Klotho* mutant mice compared to wild-type and *Klotho* heterozygous mice (Fig 5A–D). Regional overlap of Aβ with Iba-1 was observed in the GCL and NFL, while Aβ co-localized with GFAP was detected in the GCL, NFL, and OPL (Fig 5A, C).

## Discussion

In the present study, we found the abnormal ERG display in the *Klotho* null mutant mice. Both a-wave and b-wave amplitudes were reduced in the absence of Klotho function, compared with those of the littermates (Fig 1B and C). The ERG a-wave and b-wave reflect the function of photoreceptors and INL, respectively, and the latter also reflects the activities of amacrine, bipolar, horizontal, and Müller cells [38]. Our ERG data implicated that *Klotho* null mutation had impaired photoreceptors and INL functions in the retina from 8 weeks of age.

We also performed the H&E (Hematoxylin-eosin) staining to analyze the morphology and quantify the changes of retina layer thickness among the three genotypes. However, no significant difference in the morphology, thickness, and number of photoreceptor nuclei in ONL was observed among the three genotypes at 8 and 10 weeks of age. Also, we did not detect the significant difference in cell death via TUNEL staining among the three genotypes at 8 and 10 weeks of age. To further explain the impaired a-wave and b-wave response in ERG recording caused by *Klotho* null mutation, immunostainings of opsin, rhodopsin, calbindin, and Tuj1 were conducted to investigate the underlying mechanism that might cause the abnormal ERG results. The opsin and rhodopsin expression were located in the cone and rod cells, respectively, indicating a normal inception of photoreceptor transduction, which is reflected as a-wave in ERG recording [38]. Calbindin can be used to identify amacrine, horizontal, and bipolar cells in INL [43]. Thus, we utilized calbindin to investigate the effects of *Klotho* null mutation to cells in INL which are connectively involved in the neurotransmission following photoreceptor activation as reflected by b-wave in ERG recording [38,44]. Also, Tuj1 can be used to locate the RGC [41]. However, the results exhibited no significant difference in opsin, rhodopsin, calbindin, and Tuj1 expression among the three genotypes at 8 and 10 weeks of age. Thus, our present data did not show an evident impairment of photoreceptor, INL, and GCL with *Klotho* null mutation up to 10 weeks of age.

Since no evident loss of opsin and rhodopsin, nor was a decrease of RGC and INL neurons observed, we hypothesized a degenerative process with accumulation Aβ and pTau that might have occurred along with the abnormal ERG display, which had been reported in AD animal models and patients [45–47]. Therefore, we investigated whether the *Klotho* null mutation would result in Aβ and pTau deposition in the retina. Our immunostaining data demonstrated evident Aβ and pTau deposition in OPL, IPL, GCL, and NFL of the *Klotho* null mutant mouse retina at 10 weeks of age (Fig 4A–D), coherent with the previously reported Aβ and pTau accumulation in the AD mouse retina [16,48,49].

Previous literature in the AD mouse model had shown decreased a-wave and b-wave amplitude without increase of implicit time in ERG display, and no evident cell loss and disorganization were found in the retina of the AD mouse model [32]. This scenario means that Aβ or pTau accumulation may result in impaired retinal function without neuronal degeneration. Interestingly, there are some evidences indicated that *Klotho* upregulation can attenuate the Alzheimer’s disease-related pathology including memory deficiency, amyloid beta accumulation, and tau phosphorylation [9,50]. Besides, accumulating evidences have related a resemblance between the retina and the brain in AD patients and animal models [45–47], particularly the accumulation of Aβ and pTau [16,22,51]. Comparatively, our present data showed Aβ and pTau deposition in the retina in the absence of Klotho function, which is in agreement with previously reported abnormal ERG display without evident neuronal degeneration.

Since gliosis represents another major hallmark of early neuronal damages, we further investigated whether *Klotho* null mutation resulted in gliosis in the retina. Immunostaining results demonstrated the elevation of GFAP and Iba-1 expression of *Klotho* mutant mice at 10 weeks of age (Fig 4F, H). The activated glia cells were located in the OPL, IPL, GCL, and NFL of the retina in the *Klotho* mutant mice at 10 weeks of age, which was not observed in the wild-type and heterozygous retinas (Fig 4E, G). GFAP is a marker for macroglia cells, including astrocytes and Müller cells, and Iba-1 is a microglia cell marker. The elevation of these two markers suggested that both astrocytes and microglia cells were activated with *Klotho* null mutation.

Increment of activated macroglia cells can be a protective response toward injury. In the acute phase, the reactive Müller cells and astrocytes may release neurotrophic factors and antioxidants and increase metabolic activity to protect the retina [26,29]. Nevertheless, the chronic or uncontrolled activation of Müller cells and astrocytes will result in neurodegeneration [26,52]. For macroglia cells in AD, Müller cells and astrocytes become active with increased GFAP expression and even encompass amyloid plaques in the retina of a triple transgenic mouse model for AD (3xTG-AD) [25]. However, the decrement of GFAP expression, which is deemed as Müller cells degeneration, was observed in the retina of AD patients [34]. Thus, the consequence of the intricated remodeling of macroglia cells found in the *Klotho* mutant mice and the 3xTG-AD mice require further investigation [33]. On the other hand, the increased number and dysfunction of reactive microglia cells can also be found in the retina of AD patients and AD mouse model [32,34]. On the positive side, microglia cells can release trophic factor and cytokines that are vital for repair of neurons and their migration to the damage sites in order to prevent further degeneration. Nonetheless, in response to pathogen invasion, microglia cells may become overactivated and unregulated. For diminishing any threats, microglia cells consecutively secret cytokines including TNF-a and IL-1β, which may potentially lead to loss of neurons [31]. Previous reports indicated that Aβ or pTau deposition may be responsible for gliosis in the brain of AD patients [8,25]. A mouse study unveiled that intravitreal exposure to Aβ elicited gliosis in response to Aβ deposition in RGC, leading to RGC degeneration [53]. Gliosis may lead reduced a- and b- wave amplitude in ERG recording through impairing retinal vascular networks, causing fibrotic changes and retinal detachment, and disrupting the homeostasis of neurotransmitter in the retina [54,55]. Our present data showed that gliosis occurred in specific retina areas and co-localized with Aβ deposition, indicating that gliosis in the *Klotho* mutant retina may be due to Aβ deposition. Therefore, the gliosis caused by the *Klotho* null mutation may lead to disrupting effects to retinal homeostasis and the cells that are accountable for neurotransmission, as reflected by the abnormal ERG display found in the present study.

In summary, the present data demonstrated the pathologic features of AD such as accumulation of Aβ and pTau, gliosis and impaired function of retina without conspicuous cell loss or neurodegeneration in the *Klotho* mutant mice. These findings may reflect the degeneration in the brain, which may lead to the short lifespan of no more than 14.2 weeks with *Klotho* null mutation, as numerous reports have indicated that retina of AD patients or mouse models shares the pathological features with brain, especially Aβ deposition [15]. Moreover, under *in vivo* conditions, overexpression of *Klotho* can ameliorate cognitive deficit, reduce Aβ accumulation, and elicit autophagy activation, which is suggested to play a pivotal role for clearance of Aβ aggregates [9]. In addition, the amelioration of pTau pathology and neuronal injury are also found with overexpression of Klotho protein in the AD mouse model [10]. These previous findings and our current data have suggested the potential use of *Klotho* mutant mice as an AD mouse model.

However, based on our data in the present study, there are several limitations of our present study. First, we did not investigate the direct cause for the attenuated ERG response in the *Klotho* mutant mice, i.e., the examination of retinal synapse. However, the decreased synaptophysin, increased GAD67 have been found in the *Klotho* mutant mice at 7 weeks of age [4], suggesting the changes of the excitation/inhibition balance in the *Klotho* mutant mice. In addition, imbalance of fibroblast growth factor receptor, sodium potassium ATPase, and ion channel in the *Klotho* mutant retina had been found and been implicated to contribute to the homeostatic disruptions in the retina [4]. This imbalance may also affect ERG display. Therefore, further investigations are needed to better explain the ERG dysfunctions in the *Klotho* mutant retina.

Second, we found the accumulation of Aβ and pTau with gliosis in the retina of *Klotho* mutant mice which is corresponding to the findings in the AD animal models and patients. Nonetheless, we did not find the obvious morphological impairment in the retina of *Klotho* null mutant mice, such as thinning NFL, GCL and IPL, commonly observed in the clinical or preclinical AD patients [56]. Absence of this common retinal change may limit the use of *Klotho* mutant mice to reflect the typical retinopathy associated with AD. In contrast, the APPswe/PS1ΔE9 transgenic AD mouse model displayed Aβ deposition co-localized with gliosis, as well as reduced a- and b-wave amplitudes, without accompanying cell loss or neurodegeneration in the retina at 12–16 months of age [32]. This aligns with our findings. However, the Tg2576/Tg1 transgenic AD mouse model demonstrated not only Aβ deposition, inflammation, and gliosis, but also neurodegeneration of the GCL at 27 months of age [20]. This discrepancy could be attributed to differences in the gene expression and cellular localization of mutant APP and/or PS1 proteins, or the age of the AD mouse models [32]. We propose that the APPswe/PS1ΔE9 model may exhibit late-stage retinal neurodegeneration at older ages. In contrast, *Klotho* null mutation mice with short lifespan may serve as an appropriate model for studying early-stage AD pathology, particularly Aβ/pTau accumulation, gliosis, and retinal function impairment, rather than advanced neurodegeneration.

## Conclusion

We have identified characteristics of retinal degeneration induced by *Klotho* null mutation including retinal function impairment, aggregation of Aβ and pTau, and the glia cell activation, which were observed in AD patients but had not been reported with *Klotho* null mutation. Based on the findings from this study, the *Klotho* mutant mice may still help to pave the avenue for AD research, if the effects of *Klotho* null mutation on the optic nerve and the brain can be further unveiled in future researches.

## Supporting information

S1 Fig Negative control of fluorescent images of Aβ, pTau, Iba-1, GFAP, and TUNEL in the retina. (A) Negative control of Aβ image which was treated under the same conditions as the study group. Autofluorescence was found in the OS. (B) Negative control of pTau image which was treated under the same conditions as the study group. Autofluorescence was found in the OS. (C) Negative control of Iba-1 image which was treated under the same conditions as the study group. Autofluorescence was found in the OS. (D) Negative control of GFAP image which was treated under the same conditions as the study group. Autofluorescence was found in the OS. (E) Negative control of TUNEL image which was treated under the same conditions as the study group.
(TIF)

S2 Fig Analysis of retina layer thickness among the three genotypes at 8 and 10 weeks of age. (A–R) Comparison of the ONL, OPL, INL, IPL, GCL, NFL, OS to GCL, RPE to GCL, and RPE to NFL thickness among the three genotypes at 8 and 10 weeks of age. The thickness of each retinal layer was measured at 400 μm, 800 μm, 1200 μm, and 1600 μm intervals away from the optic nerve head (ONH) along the superior and inferior halves. n=6 per group presented as mean± SD. Significant difference was determined by two-way ANOVA with Bonferroni multiple comparisons test, and presented as *p < 0.05, **p < 0.01, ***p < 0.001.
(TIF)

S3 Fig Number of photoreceptor nuclei in the ONL of retina among the three genotypes at 8 and 10 weeks of age. (A–D) Comparison of the number of photoreceptor nuclei in the ONL among the three genotypes at 8 and 10 weeks of age. The number of photoreceptor nuclei in the ONL was measured at 400 μm, 800 μm, 1200 μm, and 1600 μm intervals away from the optic nerve head (ONH) along the superior and inferior halves. n=6 per group presented as mean± SD. Significant difference was determined by two-way ANOVA with Bonferroni multiple comparisons test, and presented as *p < 0.05, **p < 0.01, ***p < 0.001.
(TIF)

S4 Fig Representative images of TUNEL staining and quantification of TUNEL-positive cells in the retina among the three genotypes at 8 and 10 weeks of age. (A) Images of TUNEL-positive cells (red signals). The blue dots are cell nuclei detected by DAPI. (B) Quantification of TUNEL-positive cells and comparison among three genotypes at 8 and 10 weeks of age. Scatter plots show the means and standard deviations. Significant difference was determined by two-way ANOVA with Bonferroni multiple comparisons test, and presented as *p < 0.05, **p < 0.01, ***p < 0.001, respectively.
(TIF)
