# Supplementary figures and images for: Klotho null mutation leads to retinal degeneration characterized by functional impairment, gliosis, and deposition of amyloid-beta and hyperphosphorylated tau proteins
Source: PLoS One. 2025 May 15;20(5):e0323633. doi: 10.1371/journal.pone.0323633 (PMC12080808; doi:10.1371/journal.pone.0323633)

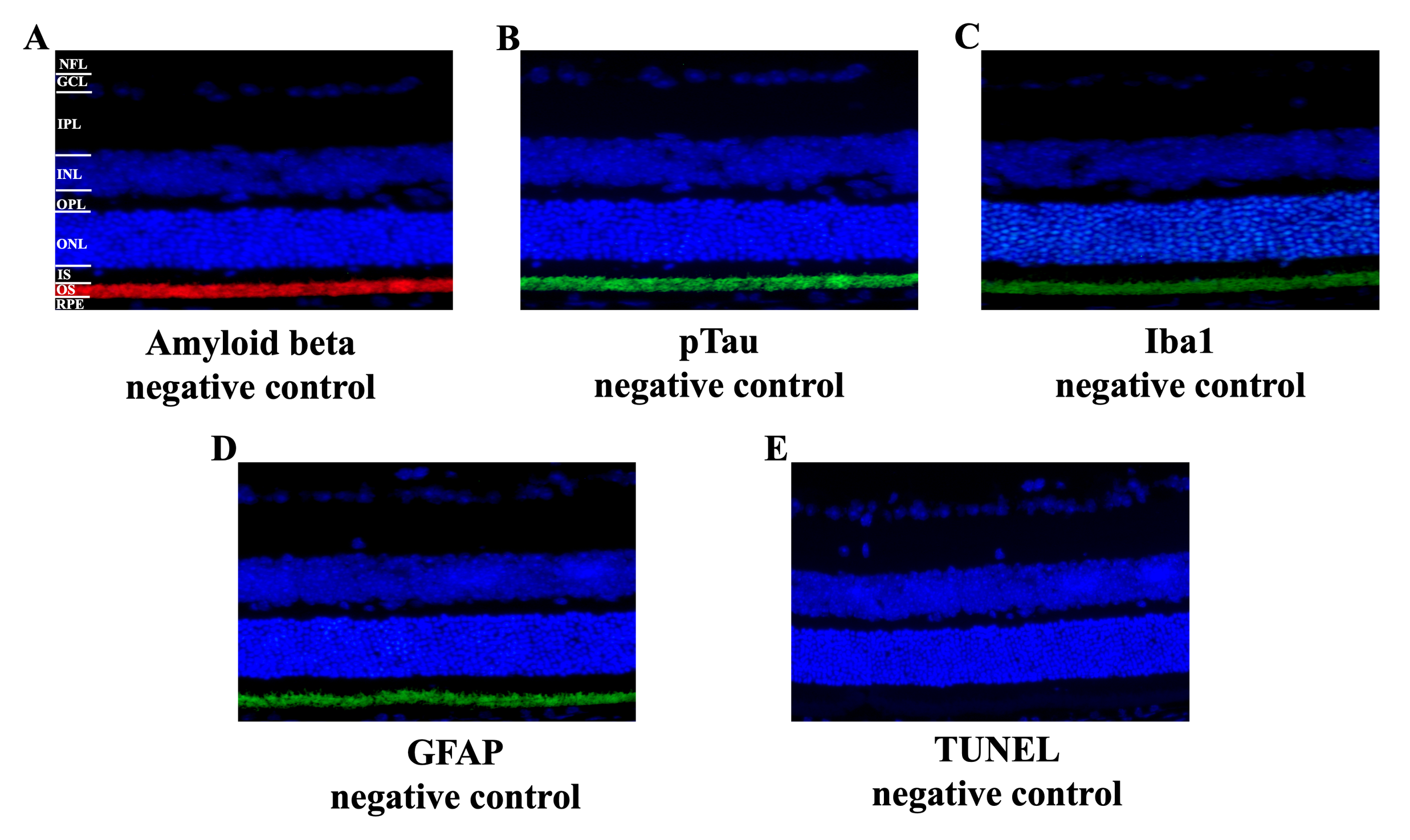

Supplement: S1 Fig — (A) Negative control of Aβ image which was treated under the same conditions as the study group. Autofluorescence was found in the OS. (B) Negative control of pTau image which was treated under the same conditions as the study group. Autofluorescence was found in the OS. (C) Negative control of Iba-1 image which was treated under the same conditions as the study group. Autofluorescence was found in the OS. (D) Negative control of GFAP image which was treated under the same conditions as the study group. Autofluorescence was found in the OS. (E) Negative control of TUNEL image which was treated under the same conditions as the study group. (TIF) [file pone.0323633.s001.tif]

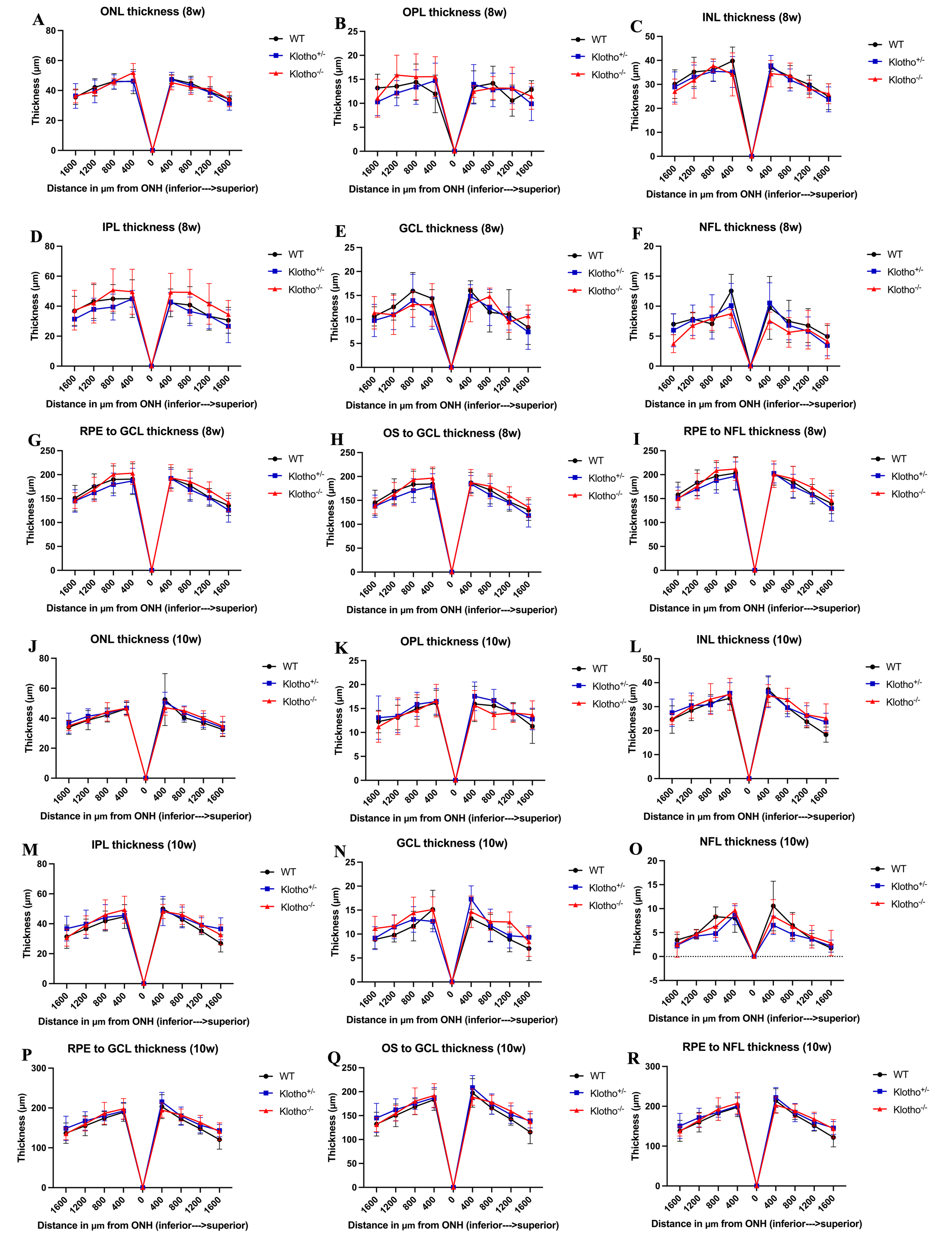

Supplement: S2 Fig — (A–R) Comparison of the ONL, OPL, INL, IPL, GCL, NFL, OS to GCL, RPE to GCL, and RPE to NFL thickness among the three genotypes at 8 and 10 weeks of age. The thickness of each retinal layer was measured at 400 μm, 800 μm, 1200 μm, and 1600 μm intervals away from the optic nerve head (ONH) along the superior and inferior halves. n=6 per group presented as mean± SD. Significant difference was determined by two-way ANOVA with Bonferroni multiple comparisons test, and presented as *p < 0.05, **p < 0.01, ***p < 0.001. (TIF) [file pone.0323633.s002.tif]

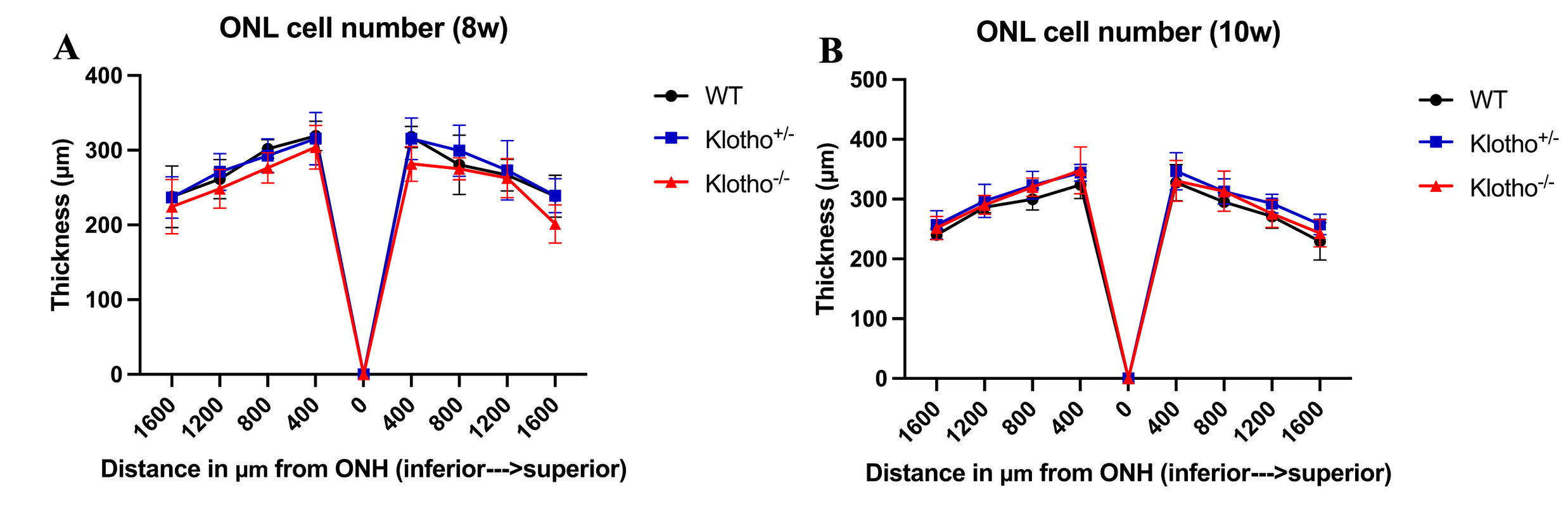

Supplement: S3 Fig — (A–D) Comparison of the number of photoreceptor nuclei in the ONL among the three genotypes at 8 and 10 weeks of age. The number of photoreceptor nuclei in the ONL was measured at 400 μm, 800 μm, 1200 μm, and 1600 μm intervals away from the optic nerve head (ONH) along the superior and inferior halves. n=6 per group presented as mean± SD. Significant difference was determined by two-way ANOVA with Bonferroni multiple comparisons test, and presented as *p < 0.05, **p < 0.01, ***p < 0.001. (TIF) [file pone.0323633.s003.tif]

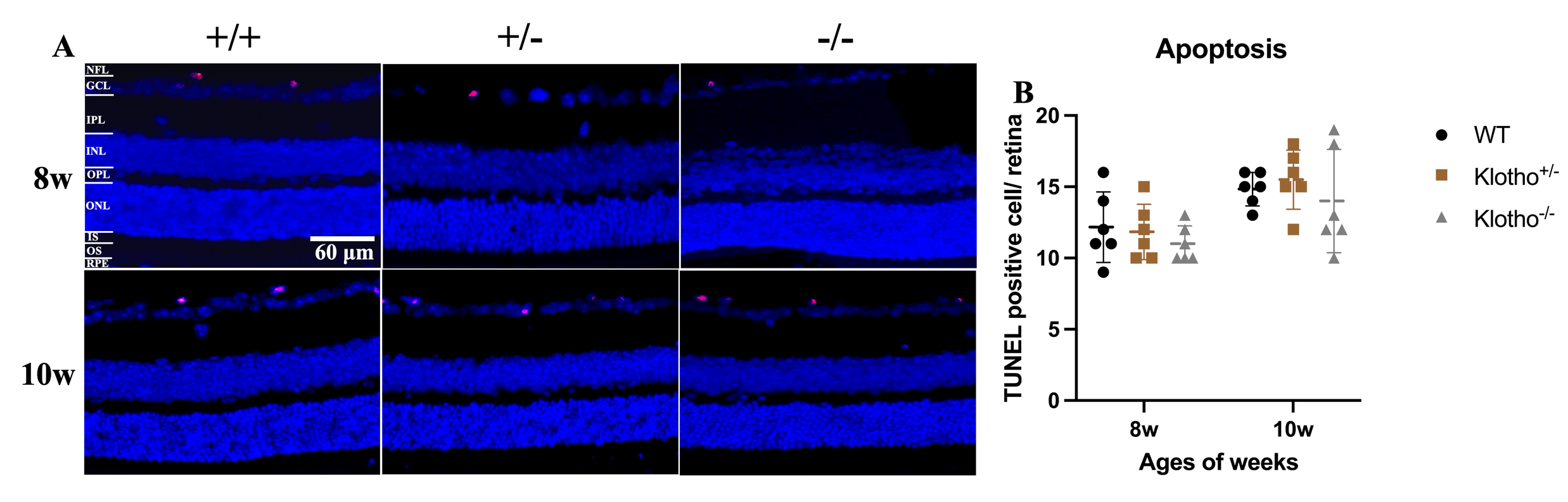

Supplement: S4 Fig — (A) Images of TUNEL-positive cells (red signals). The blue dots are cell nuclei detected by DAPI. (B) Quantification of TUNEL-positive cells and comparison among three genotypes at 8 and 10 weeks of age. Scatter plots show the means and standard deviations. Significant difference was determined by two-way ANOVA with Bonferroni multiple comparisons test, and presented as *p < 0.05, **p < 0.01, ***p < 0.001, respectively. (TIF) [file pone.0323633.s004.tif]
